# Childhood Trauma, Depression, and Suicide risk in Migrant Psychiatric Inpatients: A Cross-Sectional Study

**DOI:** 10.1192/j.eurpsy.2026.11638

**Published:** 2026-07-31

**Authors:** I. Berardelli, S. Sarubbi, L. Polidori, P. de Roberto, G. Di Biase, E. Rogante, M. Pompili

**Affiliations:** 1 Department of Neurosciences, Mental Health and Sensory Organs, Faculty of Medicine and Psychology, Suicide Prevention Centre, Sant ‘Andrea Hospital, Sapienza University of Rome; 2 Departement of Mental Health, ASL ROMA 1; 3 Psychiatry Residency Training Program, Faculty of Medicine and Psychology, Sapienza University of Rome, Psychiatry Unit, Sant’Andrea Hospital, Rome, Italy

## Abstract

**Introduction:**

International migration exposes individuals to pre-, peri-, and post-migration adversities that heighten vulnerability to mental disorders and suicidality. Global evidence shows high rates of depression and PTSD among migrants, while non-fatal suicidal behaviors such as ideation and attempts are more frequent than in host populations, particularly among women. These outcomes reflect complex interactions between psychopathology and social adversity. Yet, studies on childhood maltreatment and its psychopathological consequences among migrants remain scarce. The association between childhood abuse and suicidality is partly mediated by depressive and post-traumatic symptoms linking early trauma to suicide risk, further amplified by legal precarity, discrimination, and limited access to care.

**Objectives:**

To describe the sociodemographic and clinical characteristics of migrant psychiatric inpatients, including diagnosis, illness onset, hospitalizations, depressive symptoms, and suicide risk; to examine cross-continental differences in suicidal ideation, depression, and childhood maltreatment; and to test whether depressive symptoms mediate the relationship between childhood abuse and suicidal ideation.

**Methods:**

A cross-sectional bicentric study was conducted among 102 migrant psychiatric inpatients (63.7% women; age = 43.4 ± 12.9 years) from two university hospitals in Rome (Sant’Andrea, Policlinico Umberto I). Diagnoses followed DSM-5. Assessments included the Columbia–Suicide Severity Rating Scale (C-SSRS), Beck Depression Inventory–II (BDI-II), and Childhood Trauma Questionnaire (CTQ). Analyses used IBM SPSS Statistics 29 and comprised descriptive statistics, one-way analysis of variance (ANOVA), Pearson correlations, and mediation models (PROCESS Model 4; 5000 bootstrap samples; 95% confidence intervals; *p* < .05).

**Results:**

Overall, 31.2% had bipolar disorder, 25.5% major depressive disorder, and 24.5% a schizophrenia-spectrum disorder. Depressive symptoms were moderate (BDI-II = 19.0 ± 13.9); 35.3% reported suicidal ideation in the past month and 5% a recent attempt. Asian migrants showed higher suicidal ideation than Europeans (*p* = .012), and those from the Americas greater depressive severity (*p* = .042). CTQ subscales correlated with depression (*r* = .24–.39, *p* < .05) and suicidal ideation (*r* = .30–.46, *p* < .01). Depression fully mediated emotional-abuse effects on suicidal ideation and partially mediated physical and sexual abuse.

**Conclusions:**

Among migrant psychiatric inpatients, depressive symptoms mediated the link between childhood abuse and suicidal ideation, underscoring the role of mood dysregulation in trauma-related suicidality. Cross-continental differences suggest culturally shaped distress patterns, highlighting the need for trauma-informed, culturally sensitive prevention strategies emphasizing early detection of childhood trauma and depression in migrant mental health care.

**Disclosure of Interest:**

None Declared

